# Modeling the spatiotemporal dynamics of industrial sulfur dioxide emissions in China based on DMSP-OLS nighttime stable light data

**DOI:** 10.1371/journal.pone.0238696

**Published:** 2020-09-10

**Authors:** Yanlin Yue, Zheng Wang, Li Tian, Jincai Zhao, Zhizhu Lai, Guangxing Ji, Haibin Xia

**Affiliations:** 1 Key Laboratory of Geographic Information Science, Ministry of Education, East China Normal University, Shanghai, China; 2 Institute of Science and Development, Chinese Academy of Sciences, Beijing, China; 3 Jinan Experimental School of East China Normal University, Jinan, Shandong, China; 4 School of Business, Henan Normal University, Xinxiang, Henan, China; George Mason University, UNITED STATES

## Abstract

Due to the rapid economic growth and the heavy reliance on fossil fuels, China has become one of the countries with the highest sulfur dioxide (SO_2_) emissions, which pose a severe challenge to human health and the sustainable development of social economy. In order to cope with the serious problem of SO_2_ pollution, this study attempts to explore the spatial temporal variations of industrial SO_2_ emissions in China utilizing the Defense Meteorological Satellite Program’s Operational Linescan System (DMSP-OLS) nighttime stable light (NSL) data. We first explored the relationship between the NSL data and the statistical industrial SO_2_ emissions at the provincial level, and confirmed that there was a positive correlation between these two datasets. Consequently, 17 linear regression models were established based on the NSL data and the provincial statistical emissions to model the spatial-temporal dynamics of China’s industrial SO_2_ emissions from 1997 to 2013. Next, the NSL-based estimated results were evaluated utilizing the prefectural statistical industrial SO_2_ emissions and emission inventory data, respectively. Finally, the distribution of China’s industrial SO_2_ emissions at 1 km spatial resolution were estimated, and the temporal and spatial dynamics were explored from multiple scales (national scale, regional scale and scale of urban agglomeration). The results show that: (1) The NSL data can be successfully applied to estimate the dynamic changes of China’s industrial SO_2_ emissions. The coefficient of determination (R^2^) values of the NSL-based estimation results in most years were greater than 0.6, and the relative error (RE) values were less than 10%, when validated by the prefectural statistical SO_2_ emissions. Moreover, compared with the inventory emissions, the adjusted coefficient of determination (Adj.R-Square) reached 0.61, with the significance at the 0.001 level. (2) During the observation period, the temporal and spatial dynamics of industrial SO_2_ emissions varied greatly in different regions. The high growth type was largely distributed in China’s Western region, Central region, and Shandong Peninsula, while the no-obvious-growth type was concentrated in Western region, Beijing-Tianjin-Tangshan and Middle south of Liaoning. The high grade of industrial SO_2_ emissions was mostly concentrated in China’s Eastern region, Western region, Shanghai-Nanjing-Hangzhou and Shandong Peninsula, while the low grade mainly concentrated in China’s Western region, Middle south of Liaoning and Beijing-Tianjin-Tangshan. These results of our research can not only enhance the understanding of the spatial-temporal dynamics of industrial SO_2_ emissions in China, but also offer some scientific references for formulating feasible industrial SO_2_ emission reduction policies.

## Introduction

Sulfur dioxide (SO_2_) is one of the main pollutants in the atmosphere, which is an important indicator to measure whether the atmosphere is polluted [1]. SO_2_ is released when burning materials containing sulfur, which is found in all types of coal and oil across the world in varying proportions. As an acidic and toxic gas, SO_2_ leads to global acid rain, visibility degradation, destroy of terrestrial and aquatic ecosystems and dangerous impacts on the human health [2–6]. In brief, it causes serious losses to the whole society and the economy. SO_2_ in the atmosphere can be emitted from both natural and anthropogenic sources [7]. Natural SO_2_ sources mainly come from oxidation of biogenic dimethyl sulfide and volcanic eruption [8]. While, the primary anthropogenic SO_2_ emission is from fossil fuel consumption, especially for power generation, and other industrial production activities [9]. It is estimated that anthropogenic emissions are the main source of global SO_2_ emissions [10, 11].

China is one of the countries with the highest SO_2_ emissions [12] and the largest area exposed to acid precipitation in the world [13], because its huge economy relies heavily on fossil fuels as an energy source [14]. In 2015, China emitted 18.591 million tons of SO_2_, of which 83.73% was industrial SO_2_ [15]. In order to reduce SO_2_ emission to mitigate the adverse impacts, a range of measures has been advanced by the Chinese government, including installing flue gas desulfurization on facilities power plants [16], increasing the proportion of non-fossil fuels [17], and carrying out a plan that the SO_2_ emission in 2020 shall reduce by 15% compared with 2015 [18]. Although the Chinese government has made tremendous efforts to control the air quality, the current situation of air pollution is still not optimistic, as for the air quality in many cities still could not meet the national air quality standards [19]. For instance, in 2017, only 99 out of the 338 cities in China met the environmental air quality standards, while 70.7% failed to achieve the national air quality standards [20]. More seriously, SO_2_ can affect the atmosphere and environment on a global scale [21, 22]. It means that studying on SO_2_ emissions in China is also of great significance to improve global environmental performance. Therefore, it is urgently necessary to explore the dynamic characteristics of SO_2_ emissions in China, for obtaining a better understanding of the current air pollution situation and formulating appropriate emission reduction policies.

Remote sensing can provide valuable data sources in the research of detecting the spatial and temporal changes of geospatial information. Numerous literatures have proved that DMSP-OLS nighttime light imagery can perform well in the detection of socioeconomic activities, such as, monitoring urban dynamics [23–26], measuring spatial distribution of population [27–30], investigating economic development [31–33], estimating power energy consumption [34–37], etc. The significant correlation between the DMSP-OLS NSL data and energy consumption has been verified [38, 39], and the NSL data has been successfully applied to fossil fuel related CO_2_ emissions [40–44] and pollutant emissions (such as PM_2.5_ emissions and nitrogen oxides emissions) [45–50]. For instance, Ghosh et al. [41] applied nighttime satellite imagery favorably to map fossil fuel CO_2_ emissions. Li et al. [45] utilized nighttime light imagery to PM_2.5_ pollution estimation in Beijing using an established model and the average precision of the estimation reached 0.796. Xu et al. [46] taking Shanghai, China as an example, verified the effectiveness of DMSP nighttime light images in predicting urban daily PM_2.5_ concentrations. Toenges-Schuller et al. [47] employed DMSP-OLS nighttime light images for detecting the global distribution patterns of anthropogenic nitrogen oxides emission. Jiang et al. [48] quantified the spatial-temporal dynamics of nitrogen oxides emissions in China utilizing a NSL based model. In summary, DMSP-OLS NSL data has been proved to be promising in monitoring fossil fuel related CO_2_ emissions and pollutant emissions. As anthropogenic SO_2_ emissions are mainly from fossil fuel combustion [51, 52], thus, there is a good potential to estimate industrial SO_2_ emissions based on NSL data. Moreover, existing researches have also demonstrated that there was a significant correlation between nighttime light data and gross domestic product of secondary industry [53, 54]. Therefore, theoretically, the industrial SO_2_ emissions can also be estimated utilizing nighttime light data. In addition, different from the bottom-up emission inventories which are usually highly uncertain and not timely updated [55], NSL data can provide spatial explicit images with high resolution in time. But there are few studies about whether and how the NSL data could be applied to estimate the spatial-temporal dynamics of industrial SO_2_ emissions. Hence, we tried to apply the nighttime light data to estimate industrial SO_2_ emissions in China.

This study aims to test the utility of modeling the spatiotemporal dynamics changes of China's industrial SO_2_ emission based on DMSP-OLS nighttime stable light data. Specifically, the major objectives of our study are: (1) confirming a positive correlation was truly existed between the industrial sulfur dioxide emissions and DMSP-OLS NSL data, (2) building models to investigate China's industrial SO_2_ emission utilizing the NSL data, and evaluating the estimation accuracy using statistical emissions and emission inventory data, respectively, (3) exploring the spatiotemporal distribution characteristics of China's industrial SO_2_ emission from three different scales on the basis of the NSL-based estimation results, and putting forward some suggestions on industrial SO_2_ emission mitigation, accordingly.

## Study area and data

### Study area

**Fig 1:**
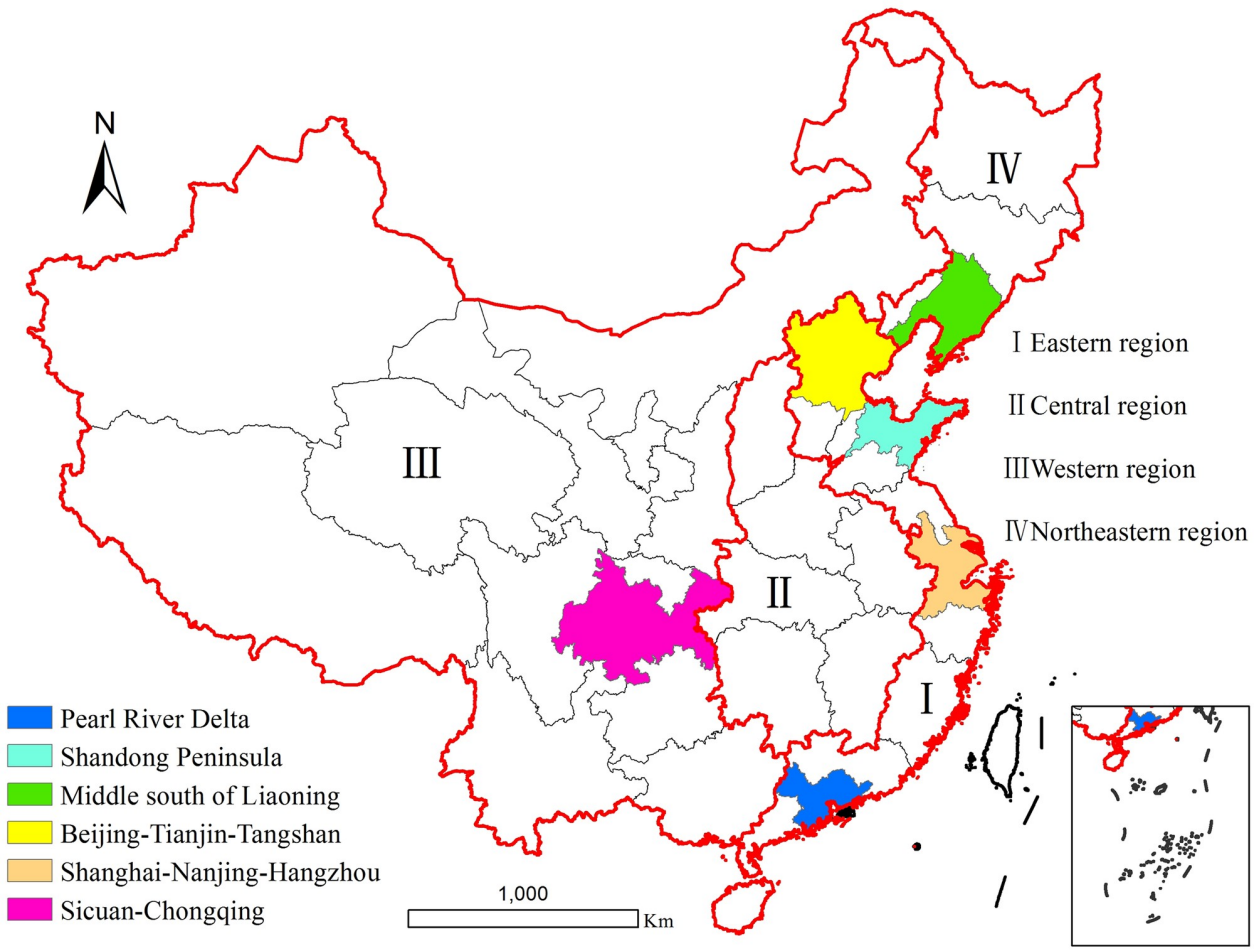
Study area.

In order to learn more about the spatiotemporal variations and changes of industrial SO_2_ emissions in China for 1997–2013, the research area was determined by three different administrative levels (Fig 1). National scale is the first administrative level. In recent years, a vast volume of SO_2_ emitted from industrial production has brought about serious atmospheric pollution. It is necessary to explore the overall situation of industrial SO_2_ emissions in the whole country firstly. Considering that the statistical industrial SO_2_ emissions data was absent in some areas (Hong Kong, Macao and Taiwan), the first level was limited to mainland China. Then, regional scale is the second level. Because of the imbalanced socioeconomic development in our country, great disparities of industrial SO_2_ emissions within different economic regions have been formed. In order to reveal the differences among regions, we divided the research area into four regions according to its geographical position and socioeconomic development level. So, Eastern region, Central region, Western region and Northeastern region were studied separately. Finally, the scale of urban agglomeration is the third administrative level. As far as we know, the population and economic growth of China were mainly concentrated in urban agglomerations, these areas contributed more to industrial SO_2_ emissions in the whole country. Consequently, it is of great significance to explore the characteristic of industrial SO_2_ emissions in urban agglomerations for reducing SO_2_ pollution. Therefore, six representative urban agglomerations were finally chosen as the third level, namely Middle south of Liaoning, Beijing-Tianjin-Tangshan, Shandong Peninsula, Pearl River Delta, Sichuan-Chongqing and Shanghai-Nanjing-Hangzhou.

### Data

There are mainly two kinds of data sets utilized in this research, namely, DMSP-OLS NSL data and statistical industrial SO_2_ emissions data. NSL data from 1997 to 2013 were derived from the National Oceanic and Atmospheric Administration’s National Geophysical Data Center (NOAA/NGDC) website (http://www.ngdc.noaa.gov/eog/dmsp/downloadV4composites.html). These NSL images measured lights on the Earth’s surface from human settlements, road networks and other sites with continuous lighting. The digital number (DN) value of the NSL imagery ranges from 0 to 63. And, the spatial resolution of the NSL images is 0.0083°(about 1 km). Due to the following two shortcomings of DMSP-OLS NSL data: (1) pixel saturation effect and (2) discontinuity and incomparability phenomenon, it is necessary to intercalibrate the data before using it. In order to intercalibrate the NSL data, Shi et al. [37] developed a modified invariant region (MIR) method, consisting of reduction of saturation effect and correction of discontinuity effect. In our study, the time series NSL data were directly intercalibrated based on the specific equations and parameters reported in the article of Shi et al.

Additionally, statistical data of industrial SO_2_ emissions including provincial statistics and prefectural statistics were derived from Statistical Yearbooks of different provinces (1998–2014) and China City Statistical Yearbook (2004, 2005, 2009, 2010, 2013 and 2014). In detail, the provincial statistics emissions were used for modeling the industrial SO_2_ emissions at 1km resolution, while the prefectural statistics emissions were used to assess the accuracy of simulation. The industrial SO_2_ emission data can be found in the S1 and S2 Files in the Supporting information section.

## Methodology

For investigating the spatial-temporal dynamics of China’s industrial SO_2_ emissions, main research procedures include: (1) correlation analysis between nighttime light images and statistical industrial SO_2_ emissions at the provincial level; (2) estimation of industrial SO_2_ emissions at 1 km resolution based on NSL data and provincial statistics emissions; (3) accuracy assessment of industrial SO_2_ emissions estimation by prefectural statistical emission data and emission inventory data; (4) multiscale analysis of the spatiotemporal characteristics of industrial SO_2_ emissions.

### Correlation analysis

Correlation analysis was employed to analyze whether there exists a significant correlation between DMSP nighttime light images and statistical industrial SO_2_ emissions or not. The formulas we used for correlation analysis can be represented as follows:

$$r_{xy}=\frac{\sum_{k=1}^{n}\left(x_{k}-\bar{x}\right)(y_{k}-\bar{y})}{\sqrt{\sum_{k=1}^{n}{\left(x_{k}-\bar{x}\right)}^{2}\sum_{k=1}^{n}{\left(y_{k}-\bar{y}\right)}^{2}}}$$ (1)

$$\bar{x}=\frac{1}{n}\sum_{k=1}^{n}x_{k}$$ (2)

$$\bar{y}=\frac{1}{n}\sum_{k=1}^{n}y_{k}$$ (3)

Where, *r*_*xy*_ is expressed as the degree of correlation between variable *x* and variable *y*, whose value ranges from -1 to 1. And the closer absolute value of *r*_*xy*_ is to 1, the stronger correlation between *x* and *y* is. In this paper, variables *x* and *y* represent DN values of DMSP nighttime light images and industrial SO_2_ emissions, respectively.

### Estimation of industrial SO_2_ emissions

Once the significant positive correlation between NSL data and statistical industrial SO_2_ emissions data is confirmed, industrial SO_2_ emission at the provincial level can be simulated using NSL data. Then, the linear regression model was performed to estimate industrial SO_2_ emissions, and the equation can be described as the following:

$$S_{P}=a\times TNSL+b$$ (4)

Where S_P_ stands for the provincial industrial SO_2_ emissions, TNSL represents the total nighttime stable light values of each province, *a* is the regression coefficient, and *b* stands for the intercept.

Considering the absence of industrial SO_2_ emissions at the pixel level, the positive correlation between NSL data and industrial SO_2_ emissions is assumed to be constant within the same province. Additionally, provincial statistical industrial SO_2_ emissions data were employed to correct the estimation models to reduce the error within a provincial unit. The formula is:

$$CS_{i}=SS_{p}\times (S_{i}÷TS_{p})$$ (5)

Where *CS*_*i*_ indicates the corrected industrial SO_2_ emissions of the *i* pixel; *SS*_*p*_ stands for the statistical industrial SO_2_ emissions of the *p* province; *S*_*i*_ is the estimated industrial SO_2_ emissions of the *i* pixel; *TS*_*p*_ represents the estimated industrial SO_2_ emissions of the *p* province.

### Accuracy assessment of industrial SO_2_ emissions estimation

It is necessary and crucial to assess the accuracy of industrial SO_2_ emissions estimation. Two indicators, the coefficient of determination (R^2^), and the relative error (RE) were often used to assess the accuracies of simulated results [56, 57]. For instance, Shi et al. [37] modeled the spatiotemporal dynamics of global electric power consumption by DMSP-OLS NSL data, and 128 samples of country-level statistical electric power consumption data from 1992 to 2012 were collected to calculate the R^2^ and RE which were employed to evaluate the estimation accuracy.

To validate the accuracy of industrial SO_2_ estimation models, R^2^, and RE are calculated:

$$R^{2}=\frac{\sum_{c=1}^{m}{\left(S_{c}-\bar{SS_{c}}\right)}^{2}}{\sum_{c=1}^{m}{\left(SS_{c}-\bar{SS_{c}}\right)}^{2}}$$ (6)

$$RE=\frac{S_{c}-SS_{c}}{SS_{c}}$$ (7)

Where, m is the total number of validation regions which was set to 263 in our research, *SS*_*c*_ stands for the statistical industrial SO_2_ emissions of the c city, $\bar{SS_{c}}$ indicates the average of *SS*_*c*_, *S*_*c*_ is the estimated industrial SO_2_ emissions of the c city. In the above three parameters, the higher R^2^ value and the lower absolute RE values indicate a higher simulation accuracy.

### Evaluation of spatiotemporal dynamics of industrial SO_2_ emissions

First of all, the average industrial SO_2_ emissions from 1997 to 2013 was calculated using the following equation to analyze the spatial pattern of industrial SO_2_ emissions:

$$\bar{S_{i}}=\frac{\sum_{n=1997}^{2013}S_{i}}{t}$$ (8)

Where $\bar{S_{i}}$ stands for the average industrial SO_2_ emissions in pixel *i* for 1997–2013, and *t* indicates the total number of years which was set to 17 in our research.

Next, the temporal variation of industrial SO_2_ emissions between 1997 and 2013 can be described by the following formula:

$$S_{i}^{tem}=S_{i}^{2013}-S_{i}^{1997}$$ (9)

Where $S_{i}^{tem}$ stands for the temporal variation of industrial SO_2_ emissions in pixel *i* from 1997 to 2013.

Then, the Natural Break method, which can maximize the differences between classes with no effect of human factors [58], was used for investigating the spatial and temporal changes of industrial SO_2_ emissions in China. In detail, the spatial variation map of industrial SO_2_ emissions was divided into five grades: low (< 3 t), relatively low (3–15 t), medium (14–37 t), relatively-high (37–70 t) and high (> 70 t). And, the temporal variation of China’s industrial SO_2_ emissions was sorted into 4 types: no-obvious-growth (< 2t), low growth (2–9 t), moderate-growth (9–22 t), and high growth (> 22 t).

## Results

### Correlation analysis results

The relationship between the DMSP nighttime light imagery and industrial SO_2_ emissions for 1997–2013 was confirmed utilizing the formulas as described in Section 3.1. And the correlation coefficients between the two from 1997 to 2013 were listed in Table 1. Obviously, the correlation coefficients over the observation period are all greater than r_0.001_ = 0.5974, which demonstrated that the correlation between these two datasets was significant at the level of α = 0.001. Based on this, 17 liner regression models were constructed to estimate industrial SO_2_ emissions. And the F values of these models are all greater than F_0.005_ (1, 29) = 9.23, revealing a statistical significance at the level of α = 0.005.

**Table 1:** Correlation analysis results.

Year	Correlation coefficient	F value
1997	0.718	30.84
1998	0.731	33.37
1999	0.714	30.20
2000	0.713	30.03
2001	0.755	38.54
2002	0.762	40.25
2003	0.728	32.72
2004	0.742	35.62
2005	0.761	39.91
2006	0.730	33.04
2007	0.741	35.28
2008	0.747	36.63
2009	0.743	35.83
2010	0.747	36.68
2011	0.738	34.64
2012	0.743	35.81
2013	0.720	31.22

#### Spatiotemporal dynamics of industrial SO_2_ emissions for 1997–2013

**Fig 2:**
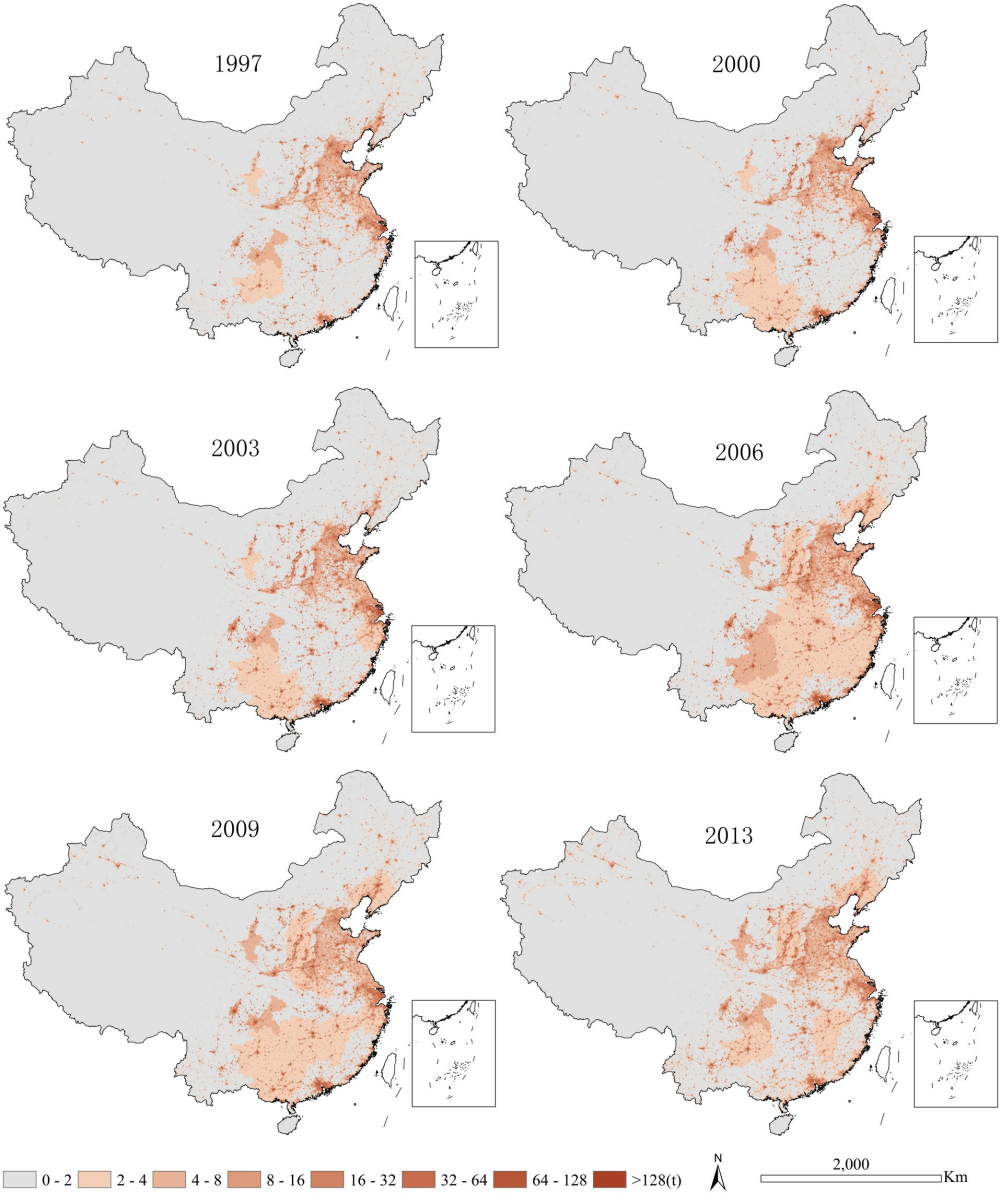
Maps of China’s industrial SO_2_ emissions for 1997–2013.

The spatial-temporal variations of China’s industrial SO_2_ emissions during the period of 1997–2013 was mapped in Fig 2. In terms of spatial distribution, industrial SO_2_ emissions in China were mainly concentrated in the eastern half of the country. Specifically, the high industrial SO_2_ emissions were clearly identified in some economically developed cities like Yangtze River Delta, Sichuan-Chongqing, and Pearl River Delta and Huang-Huai-Hai region. While, the low industrial SO_2_ emissions were largely distributed in the western and northeastern China. In terms of the temporal variations, China’s industrial SO_2_ emissions increased significantly in the initial period from 1997 to 2013 and then slowly decreased. Overall, the temporal and spatial changes of industrial SO_2_ emissions in China are significant.

#### Spatiotemporal dynamics of industrial SO_2_ emissions at national scale

**Fig 3:**
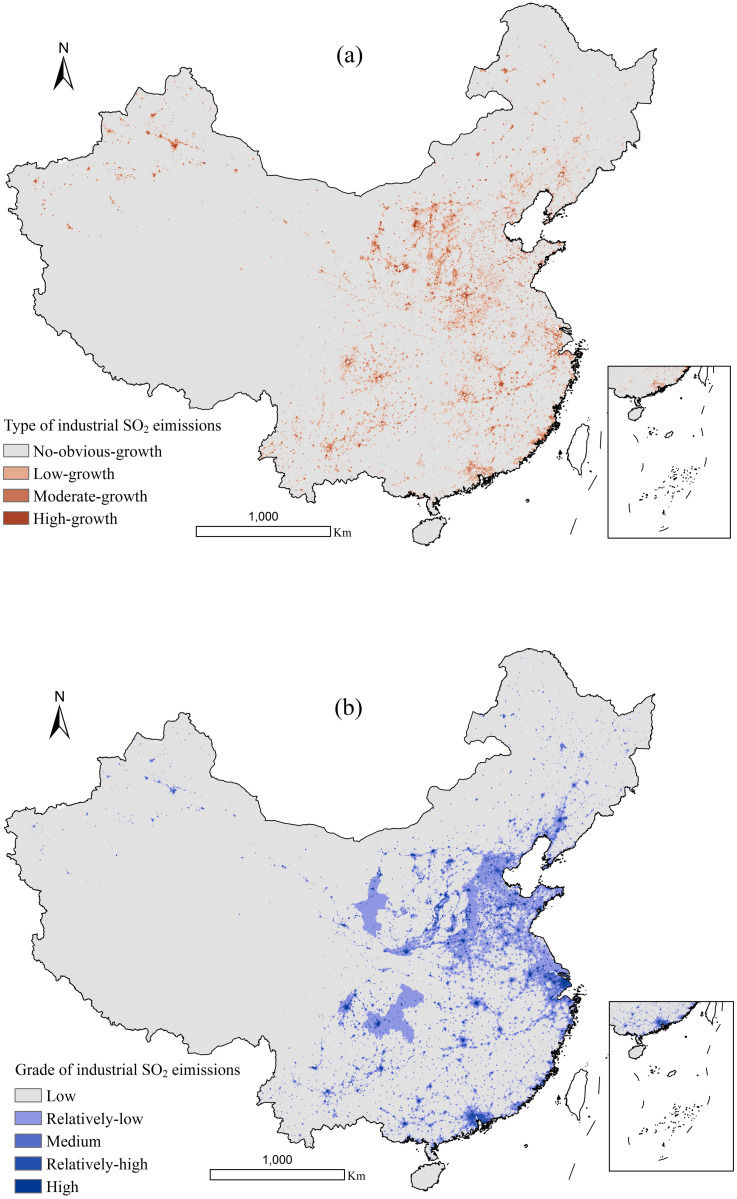
Temporal variations (a) and spatial variations (b) of China’s industrial SO_2_ emissions for 1997–2013. The non-positive growth was regarded as no-obvious growth.

**Fig 4:**
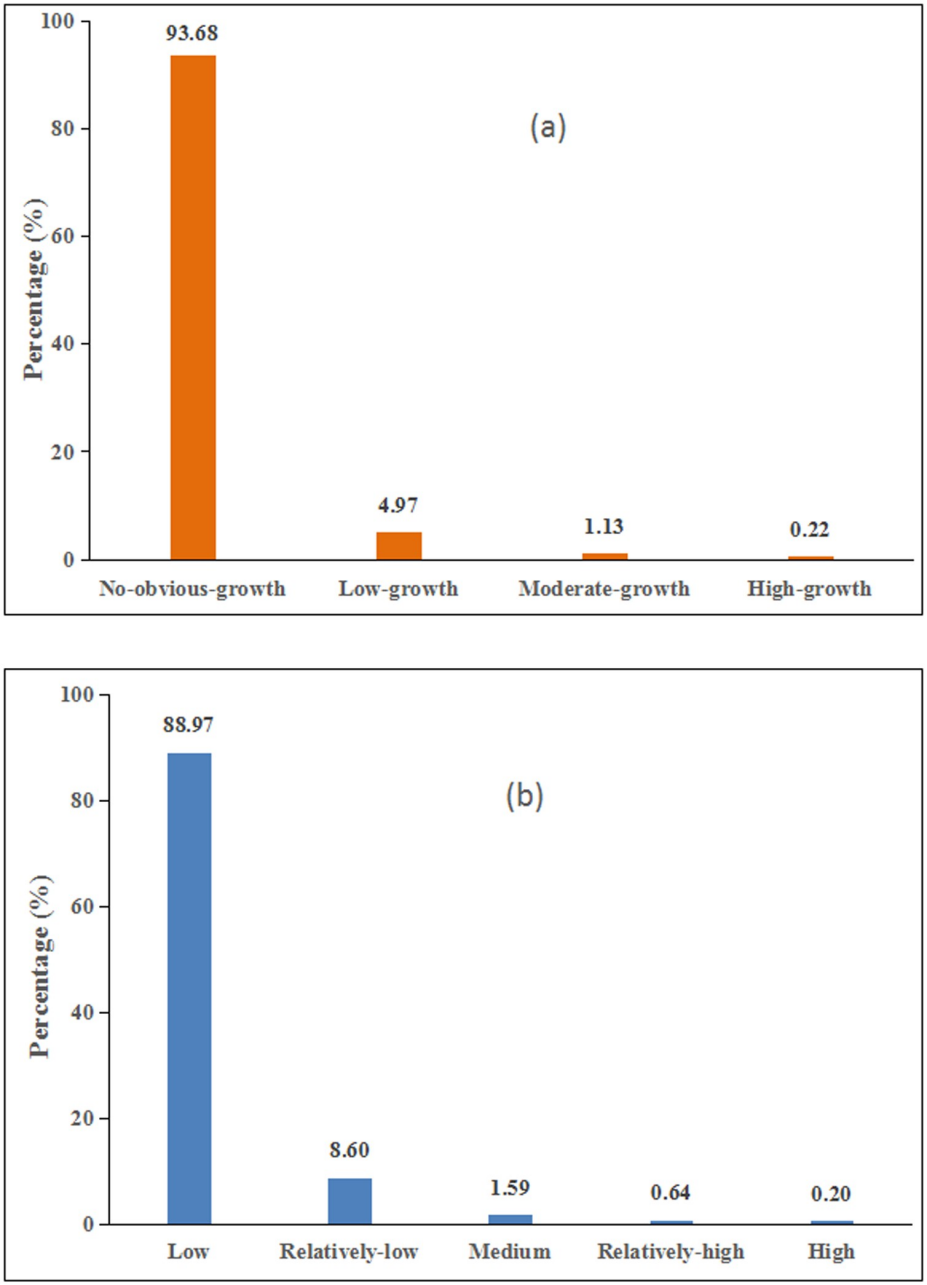
Areal percentage of each type (a) and each grade (b) in China.

Fig 3a showed the spatial distribution of the four types, which reflected the temporal variations of industrial SO_2_ emissions during the period of 1997–2013. Fig 3b described the five grades of industrial SO_2_ emissions, which indicated the spatial variations of industrial SO_2_ emissions. And, Fig 4 showed the area ratios of these four types (Fig 4a) and five grades (Fig 4b) in China. On the whole, we can find that the growth of industrial SO_2_ emissions was mostly distributed in 6.32% of China’ total areas (Figs 3a and 4a). Specifically, two types (including high-growth type and moderate-growth type) with relatively rapid growth, accounting for 1.35% of the national area, were mainly distributed in coastal areas and some metropolitan areas, including Chongqing and provincial capital cities. While, the other two types reflecting time variations of industrial SO_2_ emissions, including no-obvious-growth type and low-growth type, which occupy 93.68% and 4.97% of the total national areas respectively, were located in the Western and Northeastern regions mainly. As for spatial variations, there existed a similar variations pattern. The low and relatively-low grade of industrial SO_2_ emissions were mainly distributed in the Western region, covering 88.97% and 8.60% of the total national land, respectively (Figs 3b and 4b). Moreover, the high, relatively-high and medium grades accounted for 0.20%, 0.64%, 1.59% of the total national land, respectively, mainly distributing in the coastal and Central areas and Sichuan-Chongqing.

#### Spatiotemporal dynamics of industrial SO_2_ emissions at regional scale

**Fig 5:**
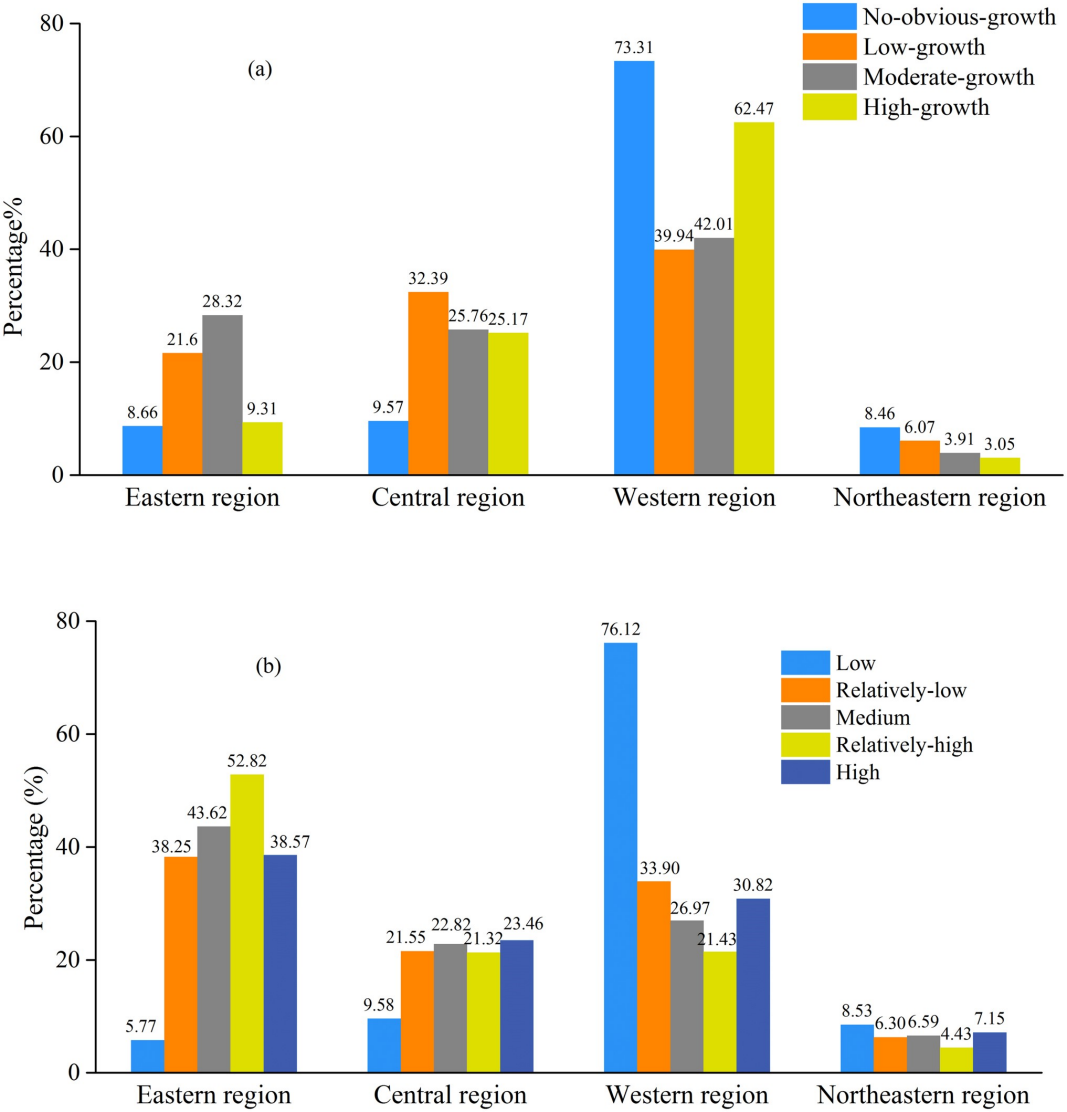
Areal percentage of each type (a) and each grade (b) in the four regions.

Fig 5 described the areal percentage of each type and each grade in China’s four regions. According to Fig 5a, the high-growth type was mostly distributed in the Western region and Central region, occupying 62.47% and 25.17% of the total areas of that type, respectively. The moderate-growth and low-growth types were comparatively evenly located in China’s Western region, Central region and Eastern region. While, the no-obvious-growth type was concentrated in the Western region mainly, covering 73.31% of the total areas of that type. Summarily, the high-growth of the industrial SO_2_ emissions was mostly located in the Western region, followed by Central region, and the no-obvious-growth was mainly located in the Western region. Both the high-growth and no-obvious-growth of the industrial SO_2_ emissions accounted for a large share in the western region, partly due to the large size of this region. Additionally, it is notable that the distribution of these four types in the Northeastern region was relatively small. This probably resulted from its relatively small size. As for the areal percentage of each grade, 38.57% of the high grade was distributed in Eastern region, 30.82% was located in Western region, 23.46% in Central region and the rest was located in Northeastern region (Fig 5b). The relatively- high grade was largely distributed in Eastern region where accounted for 52.82% of the total areas of this grade. While, 76.12% of the low grade was located in the Western region, with a fairly small proportion distributed in the Eastern, Central, and Northeastern regions. Similar to the four types, the proportions of the five grades of industrial SO_2_ emissions in the Northeastern region were all relatively low. In summary, the high grade of industrial SO_2_ emissions was mostly concentrated in the Eastern and Western regions, while the low grade was mainly located in the Western region.

#### Spatiotemporal dynamics of industrial SO_2_ emissions at urban agglomeration scale

**Fig 6:**
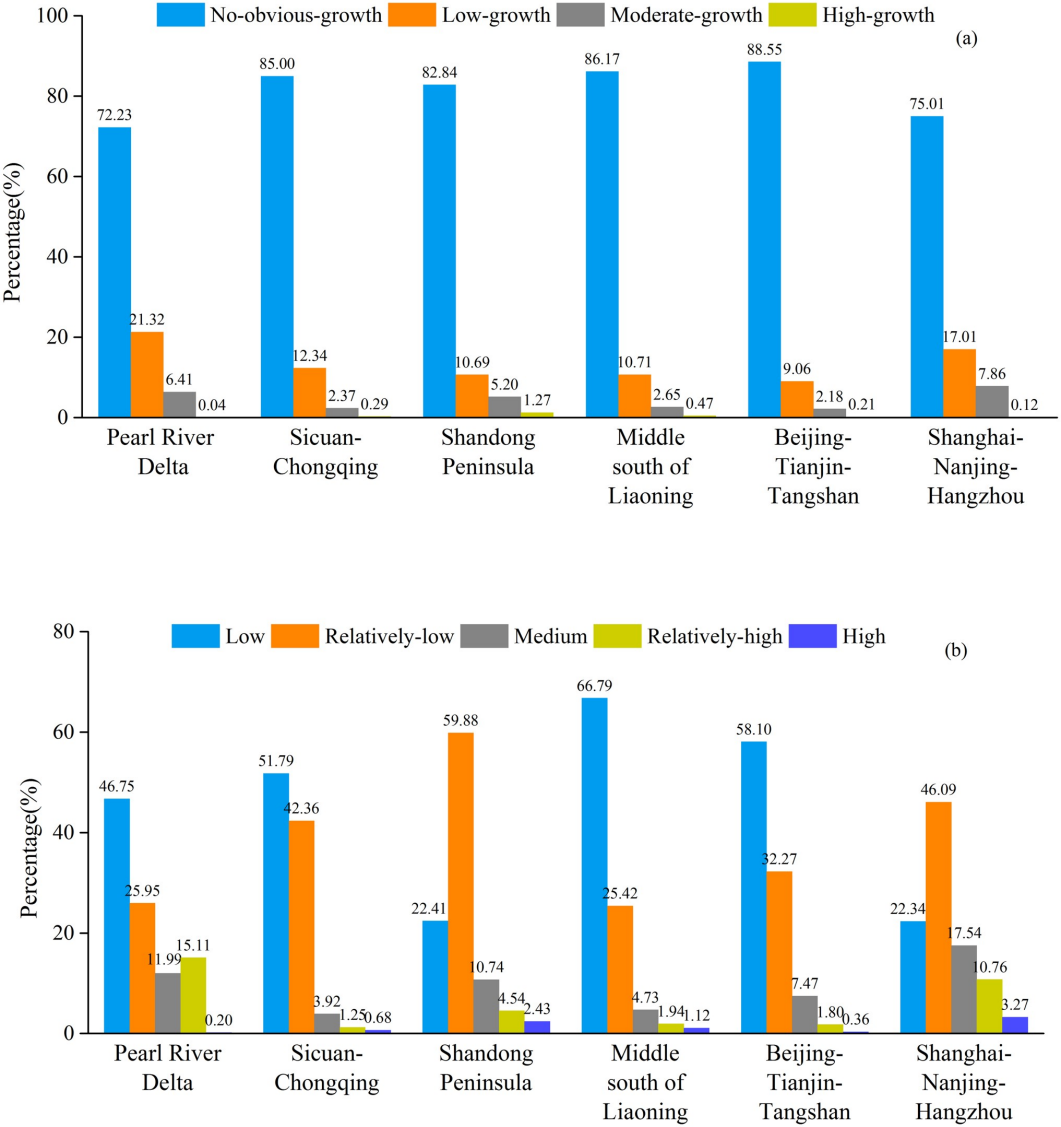
Areal percentage of each type (a) and each grade (b) within the six urban agglomerations.

The representative six urban agglomerations covered 7.68% land area of this country, but contributed 35.33% of China’s industrial SO_2_ emissions for 1997–2013. In terms of the percentage of the total areas, 88.55% of Beijing-Tianjin-Tangshan and 86.17% of Middle south of Liaoning showed a no-obvious-growth type (Fig 6a). The growth of industrial SO_2_ emissions was 21.32% in Pearl River Delta and 17.01% in Shanghai-Nanjing-Hangzhou presented a low-growth type, whereas 7.86% in Shanghai-Nanjing-Hangzhou and 6.41% in Pearl River Delta described a moderate-growth type. Besides, Shandong Peninsula should be paid much more attention to, because the areal percentage of high-growth type in this urban agglomeration was the biggest, reaching 1.27 percent. To sum up, the high-growth type of industrial SO_2_ emissions was mainly located in Shandong Peninsula, while Beijing-Tianjin-Tangshan and Middle south of Liaoning showed a no-obvious-growth variation. Moreover, the low grade of industrial SO_2_ emissions in Middle south of Liaoning and Beijing-Tianjin-Tangshan were 66.79% and 58.10%, respectively (Fig 6b). In addition, 3.27% in the Shanghai-Nanjing-Hangzhou showed a high grade and 10.76% presented a relatively-high grade. 2.43% of Shandong Peninsula presented a high grade and 4.54% showed a relatively-high grade. Summarily, the high grade of industrial SO_2_ emissions was concentrated in Shanghai-Nanjing-Hangzhou and Shandong Peninsula, while Middle south of Liaoning and Beijing-Tianjin-Tangshan presented a low grade.

## Discussion

### Accuracy evaluation of industrial SO_2_ emissions estimation

**Fig 7:**
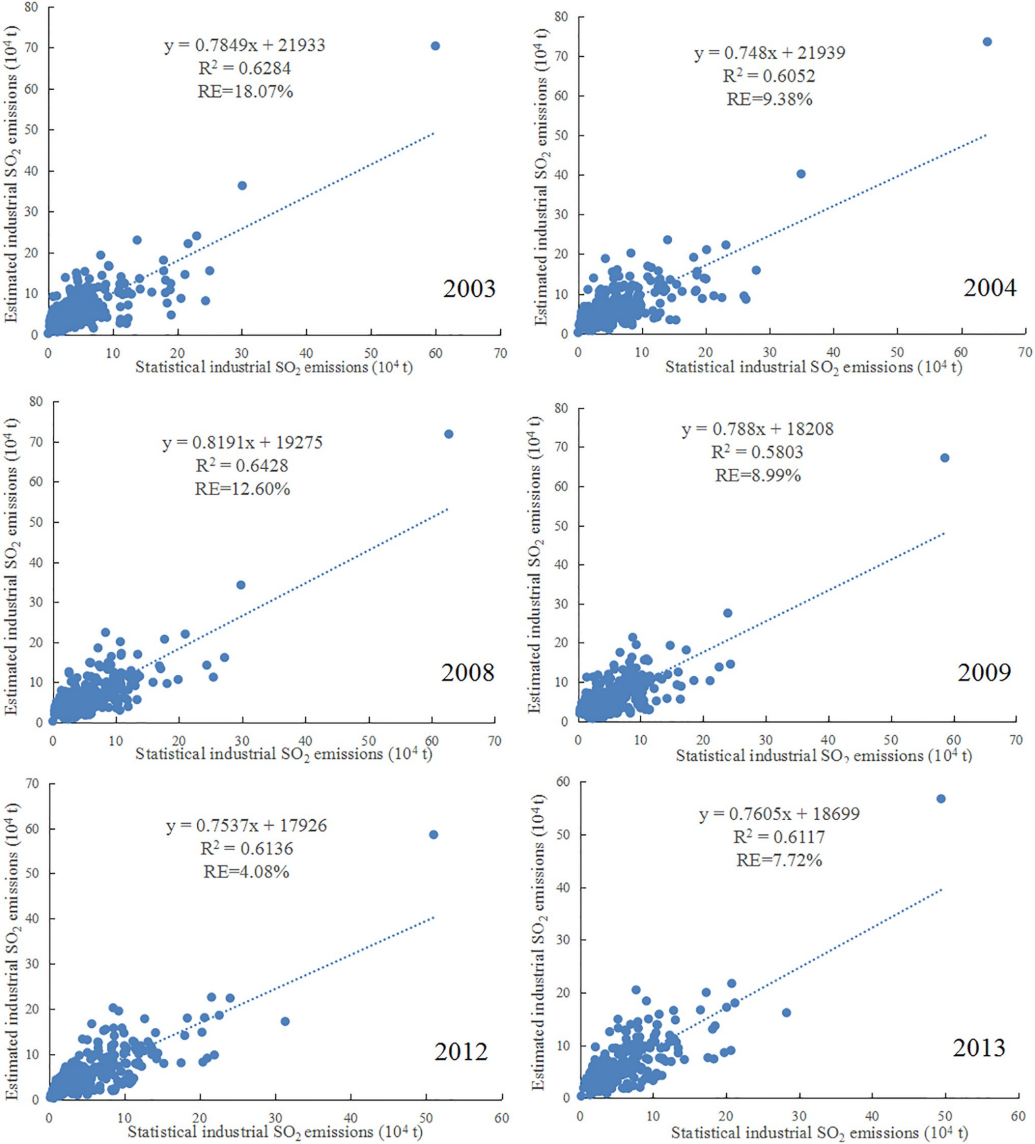
Validation scatters between the estimated and statistical industrial SO_2_ emissions.

Since we used the provincial statistical data to estimate the industrial SO_2_ emissions, it is reasonable and reliable to assess the accuracy of the estimation models utilizing the industrial SO_2_ emission data at the prefectural level. Based on data availability, statistical industrial SO_2_ emissions of 263 cities were selected to validate the estimated industrial SO_2_ emissions in 2003, 2004, 2008, 2009, 2012 and 2013. Accordingly, two indicators, R^2^ and RE were calculated to reflect the accuracy results (Fig 7).

It can be found that the minimum coefficient of determination (R^2^), was approximately 0.58, and all the other R^2^ values were higher than 0.6. Additionally, the figure also reflected that the RE was 18.07% for 2003, 9.38% for 2004, 12.60% for 2008, 8.99% for 2009, 4.08% for 2012 and 7.72% for 2013, respectively. In other words, the maximum relative error (RE) was 18.07%, and most of the RE values were lower than 10 percent. The results of our study are acceptable in comparison with the previous researches [49, 50, 57, 59]. Ji et al. [49] estimated China’s PM_10_ emissions using DMSP-OLS data, the estimation accuracy was validated by city-level statistical PM_10_ emissions for 1995, 2000 and 2005, and the R^2^ were 0.5217, 0.5437, and 0.5158, respectively. Zhao et al. [57] employed nighttime light datasets to simulate urban residential CO_2_ emissions in China, and the maximum RE is 23.084%, the average absolute value of RE is 12.84%.

**Fig 8:**
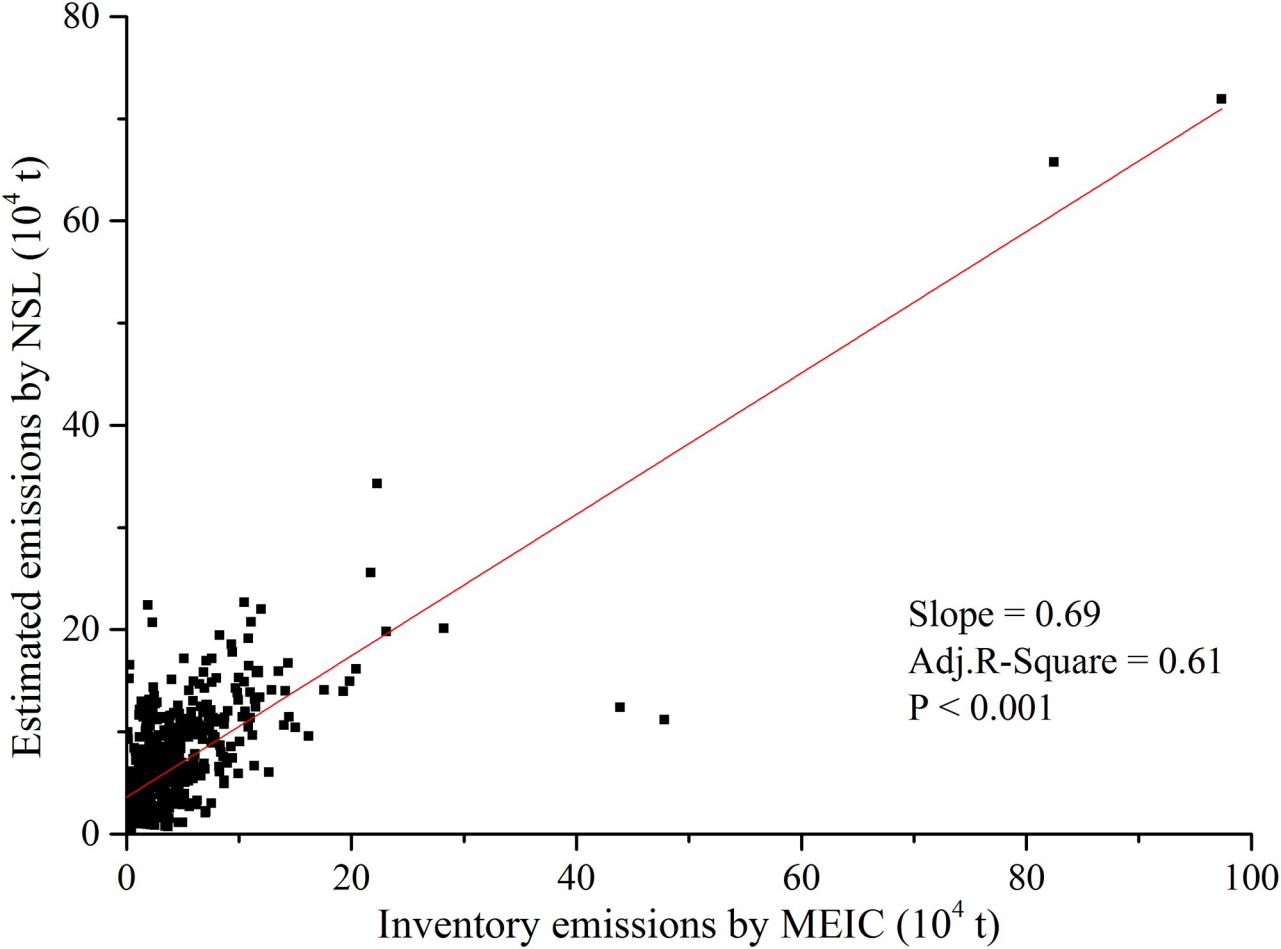
Comparison between inventory emissions by MEIC and estimated emissions by NSL at the prefectural level. Data in years of 2008, 2010; 712 samples.

Moreover, in order to further evaluate the reliability of the NSL-based estimation results, we compared the estimated industrial SO_2_ emissions with the Multi-resolution Emission Inventory for China (MEIC) database, which is a bottom-up emission inventory framework developed and maintained by Tsinghua University [60]. The emission inventory utilized in this article was downloaded from http://www.meicmodel.org/dataset-mix.html, where it is freely available for non-commercial purposes. Based on the availability of data, we compared the emissions of 356 prefecture level cities in 2008 and 2010. And the comparison results are shown in Fig 8. It can be discovered that the Adj.R-Square reached 0.61, with the significance at the 0.001 level. In other words, the DMSP-OLS NSL-based estimation results are acceptable compared with the emissions estimated by other approach.

Therefore, the accuracy evaluation results show that the industrial SO_2_ emissions in China can be modeled by NSL data. In addition, it is worth noting that the inconsistency of statistical criterion between provincial and municipal statistical data affects the simulation accuracy to a certain extent. We believe that the simulation accuracy will be improved if the availability and reliability of statistical data was enhanced.

### Suggestions for industrial SO_2_ emissions reduction

In order to reduce China’s industrial SO_2_ emissions to achieve the sustainable development of social economy, more efforts should be made to adjust, optimize and upgrade the industrial structures and enhance the energy utilizing efficiency. Considering the great differences in economic development among regions, the Chinese government should adopt differentiated mitigation strategies for different regions. For the Eastern and Central regions with higher levels of economic development, the reduction strategies of industrial SO_2_ emissions should focus on adjusting and optimizing the industrial structure. High energy-consumed industries, such as the manufactures of chemical materials and products, metal smelting and calendering, production and supply of electric power and hot power should be close down or reformed through improving the manufacturing technology, technologic process and production equipment. At the same time, the government should vigorously develop the low-energy-consuming industries such as information services, financial insurance, Internet and tourism, actively promote industrial upgrading and change the situation of heavy industrial structure. Due to the Western and Northeastern regions are mainly dominated by energy-related and heavy industries, the reduction strategies of industrial SO_2_ emissions should put more emphasis on the energy structure optimizations and energy efficiency improvement in these two regions. Since these two regions are all at the initial stages of China’s economic development, it seems to be unfeasible and unrealistic for them to alter the coal-based energy consumption structure at the present stage. But, reducing industrial SO_2_ emissions through improving energy efficiency and the performance of flue gas desulfurization facilities seems to be more feasible and effective, in a short-term period. In a long run, the Western and Northeastern regions with abundant wind and solar energy resources, can gradually develop and utilize these renewable energy sources to replace the coal-dominated energy structure. Besides, related laws and policies should be timely formulated for facilitating the industrial SO_2_ emissions reduction in the whole country. For example, extra taxation should be imposed on the industries with high industrial SO_2_ emissions in the Eastern and Central regions. While, in the Western and Northeast regions, tax breaks, loan concessions and fiscal subsidy could be granted to the industries using renewable energies or developing advanced technologies for lower industrial SO_2_ emissions. Additionally, for the six representative urban agglomerations, industrial SO_2_ emissions in Shandong Peninsula was not only higher in grade, but also more obvious in increasing trend. Therefore, more attention should be paid to industrial SO_2_ emission reduction of this urban agglomerations.

## Conclusions

In order to cope with the industrial SO_2_ pollution problems in China, this study explored the spatiotemporal dynamics of industrial SO_2_ emissions from 1997 to 2013, and put forward relevant suggestions on industrial SO_2_ emission mitigation. On the basis of proving that there was a positive correlation between the DMSP-OLS stable lights and industrial SO_2_ emissions, we tried to utilize NSL data to simulate industrial SO_2_ emissions. By building linear regression models, we estimated China's industrial SO_2_ emissions at 1 km resolution from 1997 to 2013, and evaluated the NSL-based estimation results. The accuracy evaluation results showed that the NSL-based estimation results were acceptable. Eventually, we investigated the spatiotemporal dynamic changes of China's industrial SO_2_ emissions from three different scales, and proposed corresponding reduction suggestions for industrial SO_2_ emissions. The estimation results apparently exhibited that the distribution of industrial SO_2_ emissions differed greatly during the investigation period. Specifically, the high growth type of industrial SO_2_ emissions was mainly distributed in the Western region, Central region, and Shandong Peninsula, while the no-obvious-growth type was concentrated in Western region, Beijing-Tianjin-Tangshan and Middle south of Liaoning. And, the high grade was concentrated in Eastern China, Western region, Shanghai-Nanjing-Hangzhou, and Shandong Peninsula, while the low grade mostly located in Western region, Middle south of Liaoning and Beijing-Tianjin-Tangshan. Seeing that the spatio-temporal changes of industrial SO_2_ emissions in different regions varied greatly, reduction strategies in Eastern China and Central China should put emphasis on industrial restructuring, while in Western China and Northeastern China, more attentions should be paid to optimize the regional energy structure and improve the energy utilization efficiency.

The findings of our research can not only contribute to comprehensively comprehend the regional differences of spatiotemporal industrial SO_2_ emission dynamics at the multiple scales, but also give some scientific references for formulating feasible industrial SO_2_ emission reduction policies. But, there are limitations that are worth mentioning. First, the provincial statistical industrial SO_2_ emissions which were used to model the distributions of China’s industrial SO_2_ emissions, may be distorted due to the inconsistent statistical caliber and artificial error. Second, although the linear regression model established in this study based on NSL data, was proved to be an effective means to estimate industrial SO_2_ emissions, there indeed exist errors with NSL data as the only index in the simulation models. In order to improve the simulation accuracy, other indicators (such as economic development, industrial structure, land use data, etc.) should also be taken into account when establishing estimation models. In addition, other simulation methods (like panel data analysis, exponential model, and logarithmic model, etc.) should also be tried and compared to determine which model will be better. Third, as for the spatiotemporal dynamics of industrial SO_2_ emissions varied greatly from different regions, the driving mechanism of industrial SO_2_ emissions in China should be detected in a follow-up study.

## Supporting information

S1 File Original data of industrial SO_2_ emissions of 31 provinces in China from 1997 to 2013. (XLS)

S2 File Original data of industrial SO_2_ emissions of 263 cities in China in 2003, 2004, 2008, 2009, 2012, and 2013. (XLS)
